# Effect of hyaluronic acid dermal fillers for mid-face volume enhancement: A systematic review and meta-analysis

**DOI:** 10.6026/973206300210713

**Published:** 2025-04-30

**Authors:** Pranali Prashant Pawar, Swapna Nayan, Gokul Venkateshwar, Tejas Raut, Shivani Sharma, Sanaya Seth

**Affiliations:** 1 Department of Oral and Maxillofacial Surgery, DY Patil University School of Dentistry, Navi Mumbai, Maharashtra, India

**Keywords:** Aesthetic medicine, dermal fillers, hyaluronic acid, midface, patient safety, volume augmentation

## Abstract

Mid-face volume loss is a common concern among ageing individuals, resulting in a hollow or sunken appearance. Hyaluronic acid (HA) dermal fillers have become a popular non-surgical solution to restore lost volume and enhance facial aesthetics. Therefore, it is of interest to assess the effect of various Hyaluronic acid (HA) fillers for mid-face augmentation. Known data shows the importance of volume restoration, patient satisfaction, cosmetic improvements and safety profiles. Thus, known data help guide clinical practice and future research on Hyaluronic acid dermal fillers.

## Background:

Mid-face volume loss is one of the most prominent and noticeable signs of ageing, often resulting in a hollow or sunken appearance. This condition is particularly evident in the cheeks, under-eye areas and the nasolabial folds, which lose their youthful fullness as a person ages. Several factors contribute to this gradual volume loss, including the natural depletion of hyaluronic acid (HA), collagen and elastin, along with changes in bone structure and fat redistribution. As a result, the skin becomes more lax and less supportive, leading to the sagging of facial features. Given the increasing demand for non-invasive cosmetic procedures, many individuals seek options to restore the youthful volume and contour of their faces [1, 2]. Hyaluronic Acid (HA) dermal fillers have gained immense popularity as a non-surgical solution for facial volume enhancement, particularly in the mid-face region. HA is a naturally occurring substance in the body that plays a crucial role in maintaining skin hydration, elasticity and moisture retention. As people age, the natural production of HA declines, contributing to the loss of volume in facial tissues. HA-based dermal fillers are designed to mimic the skin's natural HA, effectively replenishing the skin's moisture and adding volume to areas affected by ageing. These fillers provide an immediate, minimally invasive solution to mid-face volume loss and can be used to enhance facial contours without the need for surgery [3, 4]. The effectiveness of HA dermal fillers has made them a popular choice for those seeking facial rejuvenation. The primary advantage of using HA fillers is their ability to restore volume with minimal downtime and few risks compared to surgical alternatives. The procedure involves the injection of the filler into specific areas of the face, targeting the cheeks, under-eye hollows and nasolabial folds. The results are typically visible immediately after the procedure and can last from several months to a year, depending on the type of HA used and individual factors such as skin type and lifestyle [5, 6]. Therefore, it is of interest to assess the effect of various Hyaluronic acid (HA) fillers for mid-face augmentation.

## Materials and Methods:

## Protocol and registration:

A meta-analysis and a systematic review were conducted by the PRISMA 2020 recommendations, the Cochrane Handbook for Systematic Reviews of Interventions, version 5.1.0 and the JBI Reviewers Manual, 4th edition. This study has been filed with PROSPERO with the code - CRD42023471179. The study answered the following PICO-formatted question: "Is there a difference in the effectiveness of various hyaluronic acid dermal fillers for midface volume augmentation in patients with mild to severe mid-face volume deficiency or static rhytides?"

## Eligibility criteria:

## Inclusion criteria:

[1] Population - Studies with patients treated with Hyaluronic acid dermal fillers having mild to severe mid-face volume deficiency or static rhytids above 18 years of age irrespective of gender, ethnicity and nationality.

[2] Intervention - Studies using different formulations of Hyaluronic acid dermal fillers for malar augmentation.

[3] Comparison - Studies using different formulations of Hyaluronic acid dermal fillers or Placebo or usual care with or without placebo for malar augmentation.

[4] Outcome - Studies giving information about Mid-face volume improvement assessed irrespective of scale used, Global Aesthetic improvement

a) Study design - Studies authored in any language that can be transcribed into English.

b) Research conducted around 1-1-2000 to 30-6-2023

c) Clinical trials encompass in-vivo probes, randomized trials, controlled trials, non-randomized trials, quasi-experimental investigations, non-experimental studies, cohort studies and cross-sectional studies.

d) Publications with full-text articles were selected.

e) Studies comparing the efficacy of various formulations of hyaluronic acid dermal fillers against hyaluronic acid dermal fillers or control or placebo groups for malar augmentation.

## Exclusion criteria:

[1] Single intervention studies without the comparative group were excluded

[2] Observational studies, Review articles, case series, in-vitro and animal research were all omitted.

[3] Studies that provide merely an abstract rather than the complete text

[4] Trials where the subjects had a history of severe medical disorders or were taking any medication that may have impacted the study's outcomes.

[5] Trails involving a combination of treatments other than Hyaluronic acid dermal fillers in the intervention group

## Literature search:

The PICOS inclusion criteria outlined in the review methodology were used to identify studies. To find possibly suitable research, two reviewers looked at the titles and abstracts. Any questions were addressed with a third reviewer.

[1] Primary outcomes measured were cephalometric anatomical landmarks.

[2] The Preferred Reporting Items for Systematic Reviews and Meta-Analyses (PRISMA) for performing a meta-analysis were used.

[3] The electronic data resources used for the comprehensive search were Cochrane Central Register of Controlled Trials (CENTRAL), MEDLINE, CINAHL, EMBASE, PsycINFO, Scopus, ERIC and ScienceDirect, with controlled vocabulary and free text keywords (Table 1).

[4] Articles published between January 1, 2000 and December 2023 was searched without regard to language.

[5] The following keywords and MeSH phrases were used in conjunction with Boolean operators in the advanced search feature.

## Screening and selection of studies:

The title and abstract of each study underwent a thorough review and critical assessment by two independent reviewers. The methods employed to apply the selection criteria were as follows:

[1] Consolidation of the searched outcomes to eliminate redundant entries.

[2] Scrutiny of titles and abstracts to discard irrelevant articles.

[3] Retrieval of the complete text of potentially pertinent articles.

[4] Compilation and organization of multiple articles stemming from the same study.

[5] Examination of the full text of articles to ascertain the extent to which they met the eligibility criteria.

[6] Establish communication with researchers, if necessary, to clarify the study's eligibility.

[7] Deliberation on the inclusion of the study and proceeding with data collection.

## Data extraction:

Two reviewers individually collected information from the studies included. Disputes were once again settled through discussion. The gathering was conducted utilizing a verified list of objects evaluated for data extraction. The main components on this list were as follows:

[1] Authors, Year and Title of the study

[2] Country

[3] Study design

[4] Sample size

[5] Age range of participants

[6] Gender

[7] Intervention

[8] Type of HA filler used

[9] Volume of filler injected

[10] Comparison

[11] Outcomes

[12] Methods of outcome assessment

[13] Conclusion and other items

Information about the publication and the study, participants' statistics, study settings, measures, comparators, measures of outcomes, study design, statistical analysis, results and all other pertinent information (including funding sources and conflicts of interest) were thoroughly extracted from every study that was included. Data extraction was carried out and thoroughly recorded in distinct Excel files for each key result.

## Assessments of the risk of bias and quality:

The quality of randomized controlled trials was assessed using the Cochrane Risk of Bias-2 (RoB-2) technique. This tool assesses the risk of bias at the study level in seven categories

[1] Randomized Sequencing Generation

[2] concealment of allocation

[3] Blinding participants and personnel

[4] Blinding the result assessment

[5] Insufficient outcome data.

[6] Partial reporting.

[7] Other prejudices.

In conducting the evaluation, each study was comprehensively assessed to determine its overall risk level, which was categorized as low, moderate, or high. This assessment involved scrutinizing individual domains and their respective criteria. A study was labelled as having a low overall risk only if all domains were found to pose a low risk. Conversely, if any domain was identified as high risk, the overall risk classification was elevated to high. Studies with uncertainties in one or more domains, without any domain categorized as high risk, were deemed to have a moderate risk. To facilitate this evaluation, RevMan (Review Manager Version 5.3) software was employed, ensuring a systematic and rigorous assessment of bias risk.

## Statistical analysis for quantitative synthesis:

Meta-analysis was conducted on the studies that provided information on similar outcomes irrespective of the type of Type of HA filler. Quantitative assessment was done using Review Manager Version 5.4

## Assessment of heterogeneity:

Clinical heterogeneity refers to variations between studies in terms of participants, therapies, comparator groups, locations and results. Methodological heterogeneity refers to research design and methodological quality (risk of bias). The I square statistic (I2) indicates how much variability in impact estimates is related to heterogeneity. I2 is the observed dispersion of findings from many studies included in a meta-analysis that is genuine rather than false. Heterogeneity was judged statistically significant at P < 0.05. The Cochrane Handbook provides a rough guidance to the interpretation of I2, which is as follows:

[1] There may be no significant heterogeneity between 0 and 30%.

[2] Moderate heterogeneity may exist between 30% and 60%.

[3] Significant heterogeneity may occur between 50% and 90%. 

[4] Significant heterogeneity exists between 75% and 100%. For I2 > 50%, the random-effects model was used.

## Results:

The initial electronic database search of PubMed/MEDLINE, Cochrane Library and DOAJ produced 137 titles, 79 of which were identified as duplicates. After assessing the abstracts, two independent reviewers selected 58 suitable titles. After careful consideration and debate, the reviewers selected 21 papers for full-text examination. There were no more articles found after manually examining the reference lists of the selected research. After pre-screening, application of the inclusion and exclusion criteria and processing of the PICO questions, 12 articles were selected for the qualitative synthesis and two for quantitative evaluation (Figure 1 - see PDF).

Twelve studies were included in this systematic review whose general characteristics are presented in Table 2. These studies were conducted in different parts of the world USA [3, 7 and 12], China [4], Germany [5, 18], Korea [6, 8, 9-10 and 14] and Canada [11]. Majority of studies were conducted in Korea. All the included studies were randomized controlled trials of split-face [5, 6, 7, 8, 9- 10, 13] and parallel design [3, 4, 11, 12 and 14]. Different types of hyaluronic acid fillers were used in different studies - LGP-HAL [3], Voluma [4], CPM-26 [5, 13], VYC 20 [5, 8 , 13], mono HA filler [6], Bio-HA filler [6], SP-HAL [7], Neuramis [8], Belotero HA filler [9], YVOc filler [10], Restylane Lyft [11], Restylane Volume [11], HARC [12], Giselleligne multilayered HA filler [14]. The conclusions of all studies indicated that HA fillers are well-tolerated and provide significant improvement in mid-face volume (Table 2).

Among the included RCTs, eight [4, 6, 7- 8, 10, 12, 13-14] studies showed low risk of bias, one [11] showed moderate risk of bias and three [3, 5 and 9] studies showed high risk of bias. In studies, Prager 2017 and Weiss 2016, information relating to randomization and allocation concealment was not mentioned contributing to a high risk of bias in these studies (Figure 2 - (see PDF) & Figure 3 - (see PDF)). Effect measures refer to statistical constructs that compare outcome data between two intervention groups. For this study, the odds ratio was used as an effect measure. Meta-analysis was conducted on studies providing data on similar outcomes at the same follow-up interval. Effect measurements are statistical constructs that compare outcome data across two intervention groups. For this investigation, the odds ratio was used as an effect metric. Meta-analysis was performed on studies that reported similar results at the same follow-up interval. In this study, quantitative synthesis for mid-face volume parameter was not possible because the values were presented as median (IQR). Hence pooled analysis of GAIS scores was done. Two studies [6, 14] were included in the assessment of GAIS scores at weeks 4, 8 and 12. At week 4, pooled score obtained was 0.15[-0.58, 0.87] indicating that the GAIS score was greater with HA filler as compared to control.

Overall, these results were not statistically significant (p>0.05) with high heterogeneity (I2=88%). At week 8, pooled score obtained was 0.27[-0.52, 1.07] indicating that the GAIS score was greater with HA filler as compared to control. Overall, these results were not statistically significant (p>0.05) with high heterogeneity (I2=92%). At week 24, the pooled score obtained was 0.32[-0.25, 0.90] indicating that the GAIS score was greater with HA filler as compared to the control. Overall, these results were not statistically significant (p>0.05) with high heterogeneity (I2=80%) (Figure 4 - see PDF).

## Discussion:

The synthesis of data from selected studies reveals promising outcomes with the use of hyaluronic acid dermal fillers for midface volume augmentation. The majority of included studies reported significant improvements in volume restoration, resulting in enhanced facial aesthetics and increased patient satisfaction. The longevity of the augmentation effect varied among different formulations, with some demonstrating sustained results over extended periods. Safety profiles were generally favorable, with only minor and transient adverse events reported in a small percentage of cases. The incidence of complications, such as bruising and swelling, was consistent with the known side effects of dermal filler procedures. However, it is crucial to acknowledge the importance of injection technique, product selection and patient selection in minimising potential risks. Notably, patient-reported outcomes, including quality of life and psychosocial well-being, were considered in several studies, providing valuable insights into the holistic impact of mid-face volume augmentation. Understanding patient perspectives is essential for tailoring interventions to individual needs and expectations. Recent studies have shown promising results.

Al-Khafaji *et al.* [21] highlighted the efficacy of hyaluronic acid fillers in achieving noticeable midface volume restoration, with improvements in facial contour and high patient satisfaction rates. Their findings also emphasized a favorable safety profile, with minimal adverse effects that were transient in nature. Similarly, Zhou *et al.* [22] supported these outcomes by reporting sustained aesthetic enhancements and positive patient-reported outcomes, including improvements in self-esteem and overall quality of life. Both studies underscore the significance of proper injection technique, appropriate product selection, and careful patient assessment in ensuring optimal results and minimizing risks. Collectively, these findings contribute to the growing body of evidence that supports the use of hyaluronic acid fillers as a reliable and safe option for midface volume augmentation.

## Conclusion:

Hyaluronic acid dermal fillers are effective and safe for mid-face volume augmentation. Clinicians can utilize these findings to enhance decision-making and improve patient outcomes. Further research is needed to assess long-term effects and patient satisfaction across diverse populations.

## Figures and Tables

**Table 1:** Search strategy and PICOS tool

**Search strategy**	
Focused Question	Is there a disparity in the efficacy of different hyaluronic acid dermal fillers used for mid-face volume augmentation between individuals with mild to severe mid-face volume deficit or static rhytides?
**Search strategy**	
Population	("Midface Volume Loss" [Text Word]) OR ("mid-face contour deficiency"[Text Word])) OR (midface volume deficiency [Text Word])) OR (mid-face [Text Word])) OR (malar [Text Word])) OR (Midface Volume Deficit [Text Word])) OR (Cheek Augmentation [Text Word]) OR ("Midface Contour Deficiencies" [Text Word]) OR ("Malar Augmentation" [Text Word]) OR ("minimally invasive plastic surgery" [Text Word]) OR ("mid-face area")) OR "static rhytides" [Text Word]))
Intervention (#1)	((Hyaluronic Acid Filler Therapy [Text Word])) OR (Large Gel Particle Hyaluronic Acid [Text Word])) OR (hyaluronic acid filler [Text Word]))) OR (hyaluronic acid dermal filler [Text Word]))
Comparisons (#2)	Same as Intervention
Outcomes (#3)	(volume restoring [Text Word] OR "Full-Facial Volume Restoration" [Text Word] OR Full-Facial Volume Restoration [Text Word])) Volumizing effect [Text Word] OR aesthetic [Text Word] OR Performance [Text Word] OR Keratinized Tissue Thickness [Text Word] OR Safety [Text Word] OR patient-reported outcomes [Text Word]))
Study design (#4)	((Randomized controlled studies [Text Word] OR randomized control trials [MeSH] OR randomized control clinical trial [MeSH]))
Search Combination	#1 AND #2 AND #3 AND #4
**Database search**	
Language	Articles in English language
Electronic Databases	PubMed/MEDLINE, Cochrane Central Register of Controlled Trials, Scopus, DOAJ
Period of Publication	Studies published between 1-1-2010 to 31-12-2023

**Table 2:** Characteristics of included studies

**Study ID**	**Place of study**	**Study design**	**Sample size**	**Age**	**Gender**	**Group 1**	**Group 2**	**Volume of injection**	**Site of injection**	**Outcomes assessed**	**Follow-up**	**Author's conclusions**
Weiss 2016 [9]	USA	RCT single blind	200 150/20	52.9+-7.6	17/183	LGP-HAL	no treatment	3.8mL	midface at the supraperiosteal to subcutaneous layer	GAIS score, FACE-Q score MMVS	week 2,4 2,4,6,8,10, 12 months	The LGP-HAL therapy is well tolerated and gives considerable improvement up to a year for the repair of midface deficits.
Li 2017 [10]	China	RCT	146 119/27	20-67	6/140	Volume	delayed treatment control	1mL	deep into the subcutaneous and/ or sub periosteal plane within the medial malar region	mid-face volume, GAIS scores, patient satisfaction	1,3,6 months	The findings of this study show that Volume injected in the subcutaneous and/or sub periosteal planes was both safe and effective for malar augmentation in Chinese patients. The safety and efficacy revealed in this investigation were equivalent to those reported in trials of persons with various ethnic backgrounds.
Prager 2017 [11]	Germany	RCT split face	45 45/45	38-66 mean 50.3+-6.95	2/43	CPM-26 26mg/mL HA	VYC 20 20mg/mL HA	2mL	Injections were at either a sub dermal or periosteal level	GAIS score	3,12,18 months	In this split-face study of patients treated with CPM-26 and VYC-20 for moderate-to-severe midfacial volume loss, the majority of subjects reported improvements at visits M3 and M12, with more than half continuing progress at M18.
Jeong 2018 [12]	Korea	RCT split face	10 10/10	32-60	0/10	mono-HA filler	bio-HA filler	0.6mL	deep dermis	GAIS score, Moires topographic system score, MRI examinations	2,4,6,8,12,24,36 weeks	Over 24 weeks, the new bi-HA was demonstrated to be as effective and safe as mono-HA for malar augmentation. Both HA fillers were safe and well tolerated, resulting in little injection site reactions.
Jones 2018 [13]	California	RCT split face	20 20/20	55.4+-10.4	0/20	SP-HAL	sterile normal saline	1mL	Intradermal microdroplet injection.	degree of cheek wrinkling and elastosis, subject satisfaction	7,14,28,90,180 days	One treatment of intradermal microdroplet injections of SP-HAL to the mid to lower cheek did not show an advantage over normal saline in alleviating clinical indications of skin wrinkling and elastosis.
Jung 2019 [14]	Korea	RCT double-blind split-face	83 83/83	35-65 mean 50.6+-7.78	19/64	Neuramis	VYC 20	1mL maximum 6mL each side	not mentioned	GAIS	0,4,12,24 weeks	Neuramis® Volume Lidocaine was not inferior to VYC-20L in tempo rarely restoring mid-face volume at 24 weeks after treatment. Both of these highly cohesive hyaluronic acid fillers can be used effectively and safely for the correction of mid-face volume loss in Asians.
Park 2019 [15]	South Korea	RCT split face	9 9/9	30-50	not mentioned	Belotero HA filler	Control fillers 1. Juvederm voluma, 2. Restylane subQ, 3. Yvoir contour	0.5mL	midface region	GAIS score. Changes in Merz scale scores	2,4,12,24 weeks	The present authors discovered the largest volumizing influence with the B filler, since in all nine cases, the greatest differences in height were documented before and following surgery when this filler was supplied.
Huh 2020 [16]	Korea	RCT split face	68 68/68	20-65	not mentioned	YVOc 22mg/mL HA	RESs 20,g/mL HA	4.0mL each side	Antero medial malar region	GAIS score, MFAS score	2,14,26,52 weeks	HA fillers used for antero medial malar augmentation maintained volume for up to 52 weeks. Furthermore, both YVOC and RESS have comparable efficacy and safety profiles.
Nikolis 2020 [17]	Canada	RCT	30 17/13	30-75	0/30	Restylane Lyft (HAL)	Restylane Volume (HAV)	2cc per session	not mentioned	MFVDS score improvement, GAIS score, patient satisfaction	week 2,4,8,16	Using a treatment approach can improve outcomes for people looking for injectable treatments for midfacial volume loss and contour abnormalities.
Jones 2021 [18]	New York	RCT parallel	210 142/68	24-80	24/176	HARC	control (Juvederm Voluma XC)	6.0mL each side	midface at the sub periosteal to subcutaneous layer inferior to the maxillary prominence	GAIS score, FACE-Q score	12,24,36,48 weeks	.At 12 weeks after the last injection, HA filler was well tolerated and as effective as the control in addressing midface fullness. Aesthetic improvement and subject satisfaction were substantial
Kerscher 2022 [19]	Germany	RCT double-blind split face	45 45/45	18-65	1/44	CPM-26	VYC 20	2mL	not mentioned	photographic assessment of upper cheek fullness, GAIS scores	1,3,6,12,18 months	At month three, CPM-26 was declared non-inferior to VYC-20 based on MAS ratings and it had a satisfactory safety and efficacy profile for midface volume augmentation. These findings remained going up to month 18
Yi 2023 [20]	Korea	RCT	102	46.31+-8.72	4/98	Giselleligne multilayered HA filler	Yvoire volume plus	not mentioned	not mentioned	MFVDS score improvement, GAIS score, operators' satisfaction	1,4,8,12,24 weeks	Giselleligne demonstrated significantly higher viscoelasticity concerning volume continuity compared to other products. Moreover, the restored volume achieved with Giselleligne lasted significantly longer.
